# Extensive-Disease Small-Cell Lung Cancer With Severe Immune-Related Adverse Events Due to Atezolizumab Maintaining a Complete Response for Two Years: A Case Report

**DOI:** 10.7759/cureus.56302

**Published:** 2024-03-16

**Authors:** Sayaka Kudo, Keiki Yokoo, Nao Tanaka, Gen Yamada, Yasuo Kitamura

**Affiliations:** 1 Department of Respiratory Medicine, Kushiro City General Hospital, Kushiro, JPN; 2 Department of Respiratory Medicine, Teine Keijinkai Hospital, Sapporo, JPN

**Keywords:** immune checkpoint inhibitors, fulminant type 1 diabetes mellitus, diabetic ketoacidosis (dka), immune-related adverse event (irae), extensive-disease small-cell lung cancer

## Abstract

A 75-year-old male with diabetes mellitus was referred to our hospital with an abnormal shadow on chest radiography, based on which he was diagnosed with extensive-disease small-cell lung cancer (ED-SCLC; cT2bN2M1a).

The first-line therapy comprised atezolizumab, carboplatin, and etoposide. After four cycles, the patient achieved complete response (CR), and maintenance therapy was initiated with atezolizumab. However, even though CR was maintained, maintenance therapy was discontinued after 16 cycles due to persistent grade 2 anorexia and fatigue. Simultaneously, the HbA1c decreased to 5.5%, and antidiabetic therapy was discontinued. Six months after the last dose of atezolizumab, the patient visited the emergency room because of anorexia, dry mouth, and fatigue. Laboratory findings were as follows: blood glucose was 668 mg/dL, glycated hemoglobin (HbA1c) was 8.8%, urine ketone was 2+, sodium (Na) was 127 mmol/L, potassium (K) was 6.5 mmol/L, creatinine (Cre) was 1.43 mg/dL, and arterial pH was 7.29. Based on these findings, his presentation was consistent with fulminant type 1 diabetes mellitus (T1DM) complicated by diabetic ketoacidosis (DKA). Regular continuous insulin and saline administration was initiated in the intensive care unit, and acidosis and electrolyte abnormalities were corrected. His C-peptide was <0.03 ng/mL. His insulin secretory capacity was considered to be depleted, and he required continuous subcutaneous insulin injections. Glutamic acid decarboxylase and insulin autoantibodies were absent. The complete response persisted without further therapy until two years since the event.

## Introduction

Two pivotal clinical trials, IMpower133 and CASPIAN, have demonstrated prolonged overall survival(OS) in patients with extensive-disease small-cell lung cancer (ED-SCLC) [1,2]. Immune checkpoint inhibitors (ICIs), platinum agents, and etoposides are considered mainstream therapies. Although some reports suggest a positive correlation between the occurrence of immune-related adverse events (irAEs) and survival in patients with non-small-cell lung cancer (NSCLC) [3], the correlation between these in ED-SCLC is unclear [4-6].

The development of type 1 diabetes mellitus (T1DM) as an irAE has rarely been reported and can be life-threatening if treatment is delayed. In particular, although "fulminant type 1 diabetes mellitus (f-T1DM)" is more common in Asians [7,8], f-T1DM has rarely been reported in Caucasian subjects [9]. However, it became frequent since immune-check point inhibitors were used for treatment.

F-T1DM has been reported four or six months after discontinuation of ICI administration in NSCLC [10]. However, there are few reports on cases of f-T1DM in patients with ED-SCLC [4]. Herein, we report a case of a patient with ED-SCLC who maintained complete response (CR) despite no anti-cancer therapy one year after the onset of f-T1DM complicated by diabetic ketoacidosis (DKA).

## Case presentation

**Figure 1:**
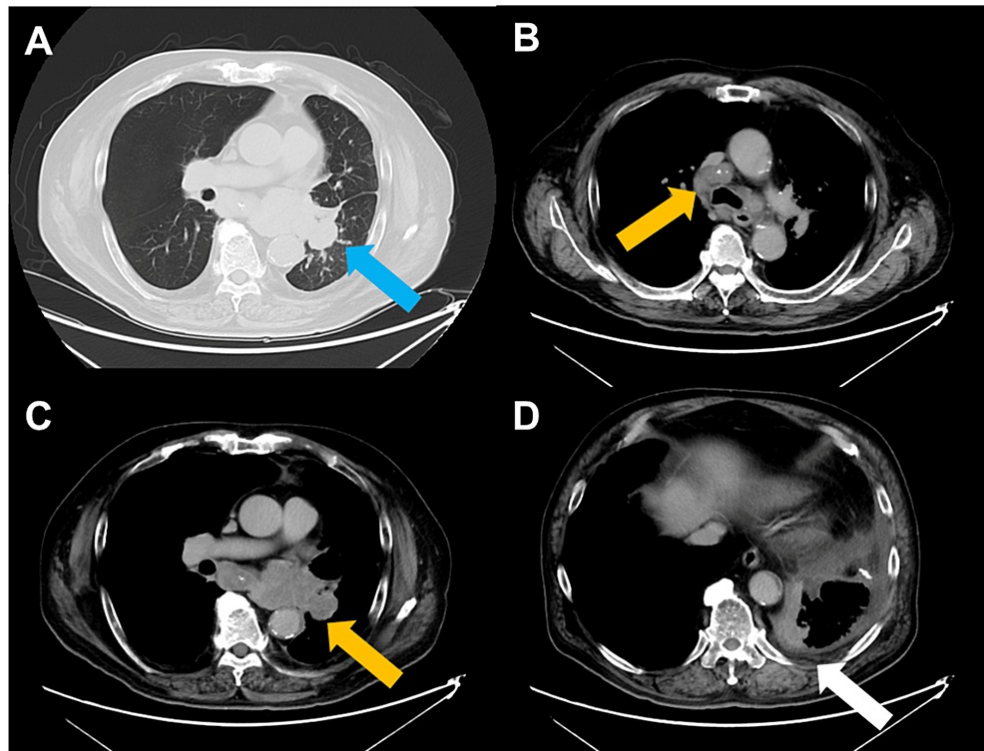
Chest computed tomography before administration of immune checkpoint inhibitor. Chest computed tomography demonstrated (A) a solitary lesion in the lower lobe of the left lung (blue arrow), (B and C) swelling of multiple hilar and mediastinal lymph nodes (yellow arrow), and (D) pleural dissemination (white arrow).

A 75-year-old male with a history of smoking (75 pack years) was referred to our hospital for dyspnea lasting for two months. The chest radiography demonstrated an abnormal shadow in the lower left lung field. Chest computed tomography (CT) revealed a solitary lesion (47 × 35 mm) in the lower lobe of the left lung, along with enlarged multiple lymph nodes in the mediastinal and hilar regions (Figures 1A-1D).

The values of specific tumor markers neuron-specific enolase (NSE) was 41.2 ng/mL, exceeding the reference range of <16.3 ng/mL and pro-gastrin-releasing peptide (Pro-GRP) was 1750 pg/mL, exceeding the reference range of <81 pg/mL, respectively. A transbronchial nodal biopsy of the mediastinal lymph node was performed from #4R and #7 for pathological examination, which revealed small cell carcinoma (SCLC). Positron emission tomography-computed tomography revealed a mass with high metabolic activity in the lower left lung, mediastinal and hilar lymph nodes, and superficial pleura in the left lung, indicating ED-SCLC; cT2bN2M1a.

**Figure 2:**
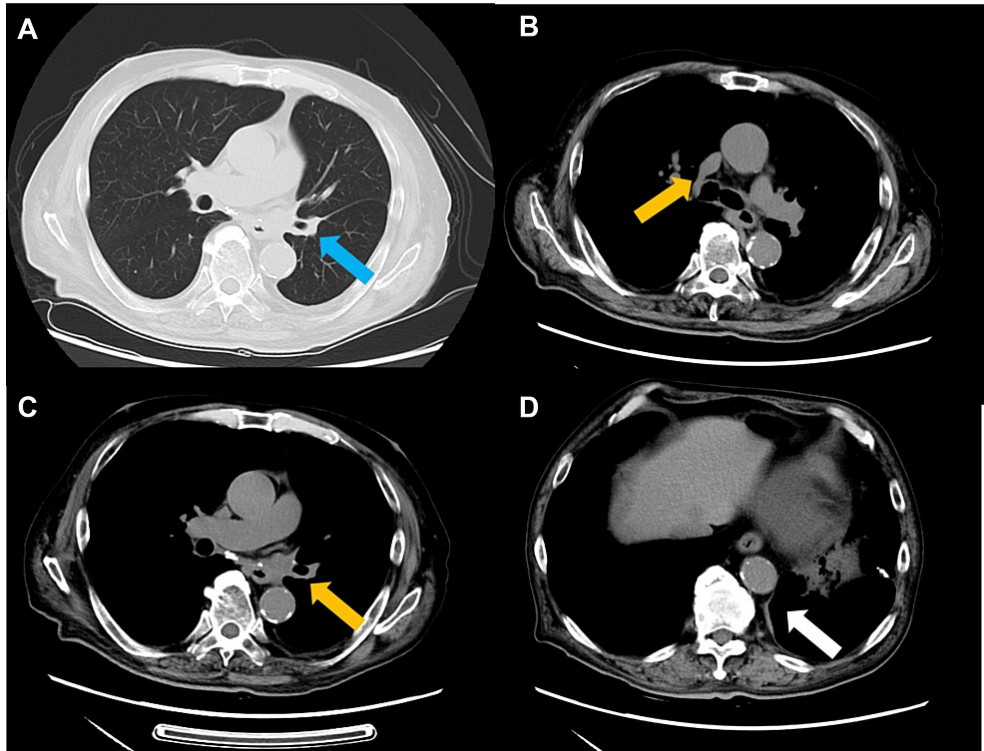
Chest computed tomography at the onset of fulminant type 1 diabetes mellitus. Chest computed tomography at the onset of fulminant type 1 diabetes mellitus demonstrated (A) shrunk primary lesion (blue arrow), (B and C) lymph nodes (yellow arrow), and (D) pleural dissemination (white arrow). A complete response was achieved.

Subsequently, first-line treatment with atezolizumab, carboplatin, and etoposide was initiated in November 2020. No adverse events were observed during the four cycles of chemotherapy and ICI. After four cycles of treatment, CR was achieved (Figures 2A-2D).

Therefore, maintenance therapy was initiated. Although CR was maintained after 15 cycles of atezolizumab therapy in January 2022, maintenance therapy was discontinued because of persistent grade 2 anorexia and fatigue. The patient was followed up with a careful imaging evaluation. Simultaneously, the HbA1c decreased to 5.6%, diabetes mellitus (DM) improved, and DM therapy was temporarily discontinued. In June 2022, the blood glucose was 135 mg/dL and the HbA1c was 5.9%. The treatment for DM was discontinued due to a stabilized level of HbA1c.

In July 2022, six months after the last dose of atezolizumab, the patient experienced anorexia exacerbation and was admitted to the gastroenterology department. He was administered glimepiride because his blood glucose was >300 mg/dL and discharged from the hospital. However, the patient visited the emergency department five days after restarting treatment for DM because of anorexia, dry mouth, and fatigue. The consciousness was clear, but the laboratory findings were as follows: blood glucose was 668 mg/dL, HbA1c was 8.8%, urine ketone was 2+, sodium (Na) was 127 mmol/L, potassium (K) was 6.5 mmol/L, creatinine (Cre) was 1.43 mg/dL (Table 1). In the present case, based on these findings, three main findings of diagnostic criteria are present, and fulminant type 1 diabetes mellitus was confirmed (Table 2) [11].

**Table 1:** Laboratory findings at the onset of fulminant type 1 diabetes mellitus and diabetic ketoacidosis. ACTH: adrenocorticotropic hormone; Alb: albumin; ALT: alanine aminotransferase; Amy: amylase; anti-GAD antibody; anti-glutamic acid decarboxylase antibody; AST: aspartate transaminase; Bas: basophil; BE: base excess; BUN: blood urea nitrogen; Ca: calcium; CK: creatine kinase; Cl: chlorine; Cr: creatinine; CRP: C-reactive protein; Eos: eosinophil; FT3: free triiodothyronine; FT4: free thyroxine; Glu: glucose; Hb: hemoglobin; HbA1c: hemoglobin A1c; HCO_3_-: hydrogen carbonate; IA-2: anti-insulinoma-associated protein-2; K: potassium; Lac: lactate; LDH: lactate dehydrogenase; Lip: lipase; Lym: lymphocyte; Mon: monocyte; Na: sodium; Neu: neutrophil; NSE: neuron-specific enolase; PaCO_2_: partial pressure of carbon dioxide; PaO_2_: partial pressure of oxygen; pH: potential of hydrogen; Plt: platelet; Pro-GRP: pro-gastrin-releasing peptide; RBC: red blood cell count; T-bil: total bilirubin; TP: total protein; TSH: thyroid-stimulating hormone; WBC: white blood cell; HCT: hematocrit

Laboratory tests	Values	Reference range
WBC	13400/μL	3900-9800/μL
Neu	91.0%	45.0-74.0%
Lym	4.4%	18.0-59.0%
Mon	4.6%	0-8.0%
Eos	0.1%	0-6.0%
Bas	0.0%	0-2.0%
RBC	4.19×10^6^/μL	4.27-5.00×10^6^/μl
Hgb	13.9 g/dL	13.5-17.6 g/dL
HCT	42.9%	39.8-51.8%
PLT	29.6×10^4^/μL	13.1-36.2×10^4^/μl
T.P.	6.0 g/dL	6.7-8.3 g/dL
Alb	3.3 g/dL	3.8-5.2 g/dL
T. Bil	0.6 mg/dL	0.3-1.2 mg/dL
AST	12 U/L	10-40 U/L
ALT	16 U/L	5-40 U/L
LDH	172 IU	124-222 U/L
ALP	270 IU	38-113 U/L
Lipase	59.5 U/L	13-55 U/L
BUN	44.3 mg/dL	8.0-22.0 mg/dL
Cre	1.43 mg/dL	0.61-1.04 mg/dL
Na	127 mEq/L	136-147 mEq/L
K	6.5 mEq/L	3.6-5.0 mEq/L
Cl	91 mEq/L	98-109 mEq/L
Ca	9.2 mg/dL	8.5-10.2 mg/dL
P	5.0 mg/dL	2.4-4.3 mg/dL
CRP	6.67 mg/dL	0-0.14 mg/dL
Blood sugar	668 mg/dL	70-109 mg/dL
HbA1c (NGSP)	8.8%	4.6-6.2%
NSE	6.9 ng/mL	<16.3 ng/mL
Pro-GRP	33.2 pg/mL	<81 pg/mL
Plasma osmolality	467 mOsm/kg	276-292 mOsm/kg
Serum Insulin	0.5 μU/mL	1.84-12.2 μU/mL
IA-2 Antibody	<0.6 U/mL	<0.6 U/mL
GAD Antibody	<5.0 U/mL	<5.0 U/mL
C-peptide (serum)	<0.03 ng/mL	0.61-2.09 ng/mL
Free T4	1.42 ng/dL	0.75-1.45 ng/dL
TSH	0.464 μIU/mL	0.61-4.23 μIU/mL
ACTH	11.20 pg/mL	7.2-63.3 pg/mL
Cortisol	21.50 μg/dL	7.07-19.6 μg/dL
Arterial blood gas analysis (room air)		
pH	7.293	7.35-7.45
PaO_2_	97.6 mmHg	80-100 mmHg
PaCO_2_	22.8 mmHg	35-45 mmHg
HCO3-	10.8 mmol/L	22-26 mmol/L
BE	-13.8 mmol/L	-2, +2 mmol/L
Lac	15 mmol/L	0.5-2 mmol/L
Urinalysis		
Glucose	2000 mg/dL	<20 mg/dL
Protein	(-)	Negative
Blood	(-)	Negative
Ketone	(2+)	Negative

**Table 2:** Criteria for definite diagnosis of fulminant type 1 diabetes mellitus. ^†^This value is not applicable to patients with previously diagnosed glucose intolerance. HLA: human leukocyte antigen; NGSP: National Glycohemoglobin Standardization Program

Fulminant type 1 diabetes mellitus is confirmed when all the following three findings are present	
1.	Occurrence of diabetic ketosis or ketoacidosis soon (approximately seven days) after the onset of hyperglycemic symptoms (elevation of urinary and/or serum ketone bodies at first visit)
2.	Plasma glucose level ≥16.0 mmol/L (≥288 mg/dL) and glycated hemoglobin level <8.7% (NGSP value) at first visit^†^
3.	Urinary C-peptide excretion <10 μg/day or fasting serum C-peptide level <0.3 ng/mL (<0.10 nmol/L) and <0.5 ng/mL (<0.17nmol/L) after intravenous glucagon (or after meal) load at onset
Other findings in fulminant type 1 diabetes mellitus	
1.	Islet-related autoantibodies, such as antibodies to glutamic acid decarboxylase, islet-associated antigen 2, and insulin, are undetectable in general
2.	Duration of the disease before the start of insulin treatment can be 1-2 weeks
3.	Elevation of serum pancreatic enzyme levels (amylase, lipase, or elastase-1) is observed in 98% of the patients
4.	Flu-like symptoms (fever, upper respiratory symptoms, etc.) or gastrointestinal symptoms (upper abdominal pain, nausea, and/or vomiting, etc.) precede the disease onset in 70% of patients
5.	The disease can occur during pregnancy or just after delivery
6.	Association with HLA DRB1*04:05-DQB1*04:01 is reported

Ten units of rapid-acting insulin were administered intravenously; however, the blood glucose was 546 mg/dL, and the K was 4.2 mEq/L. An additional 10 units of rapid-acting insulin were administered, and the patient was admitted to the intensive care unit (ICU). Continuous insulin and saline administration corrected the acidosis and electrolyte abnormalities after 18 h.

**Figure 3:**
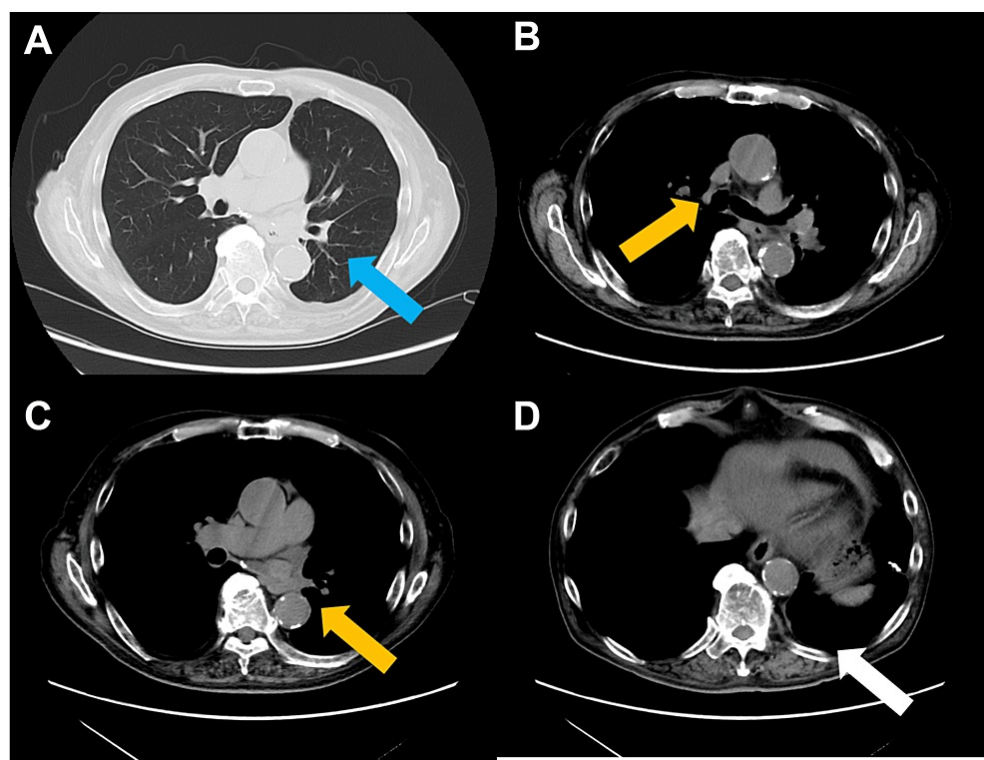
Chest computed tomography after two years from discontinuation of immune checkpoint inhibitor. Chest computed tomography demonstrated complete remission (A) primary lesion (blue arrow), (B and C) lymph nodes (yellow arrow), and (D) pleural dissemination (white arrow). A complete response was maintained.

The patient’s C-peptide was <0.03 ng/mL, his insulin secretory capacity was considered to be depleted, and insulin self-injection was initiated after discharge from the ICU. The patient was discharged from the hospital after receiving instructions on self-injection. The patient was kept under continuous observation after discharge, and he remained in CR until the latest follow-up in December 2023, two years after the last dose of atezolizumab (Figures 3A-3D).

## Discussion

This is a case of ED-SCLC with f-T1DM with DKA, a severe irAE that developed six months after discontinuation of atezolizumab administration. In clinical trials, the timing of the onset of irAEs is unclear and varies in content and timing depending on the drug used [1,2].

In NSCLC, there are reports that T1DM develops after the discontinuation of ICI administration [10,12]. It could be assumed that patients with advanced or recurrent NSCLC, even those without driver mutations, generally have a longer OS than those with ED-SCLC [13,14]. Some data suggest that the development of irAEs contributes to OS in NSCLC. In particular, prolonged prognosis has been observed when grade 2 or lower irAEs occur [3]. Therefore, patients with NSCLC should be followed up carefully for the development of irAEs, not only during the treatment period but even after the discontinuation of ICIs. However, this is not clear for small cell carcinomas. There are some reports of a relationship between irAEs and OS in the SCLC which is treated by sintilimab or combination therapy (anti-CTLA-4 and anti-PD-1/anti-PD-L1) [4,5]. However, in clinical settings, atezolizumab and durvalumab are globally used for first-line treatment of SCLC. It is unclear whether severe irAE due to atezolizumab and durvalumab contribute to OS in SCLC. A retrospective study demonstrated that no relationship exists between irAEs and survival in ED-SCLC; some patients who received second-line therapy had prolonged OS [6].

Interestingly, in the present case, CR persisted for two years after the last dose of atezolizumab without additional anti-cancer treatment after the onset of irAEs. Such a clinical course is often experienced in NSCLC. It was found that some cases of small cell carcinoma can maintain long-term CR, as in the present case, by overcoming severe irAE. Hence, some patients with ED-SCLC could have a clinical course similar to that of NSCLC.

F-T1DM is not a novel statement. Although the statement is more common in Asians, it became frequent since immune-check point inhibitors were used for treatment. The mechanism of T1DM caused by ICIs is characterized by islet β-cell destruction [15]. When the PD-1 pathway is blocked by anti-PD-1/PD-L1 therapy, not only T cells targeting cancer survive but also autoreactive T cells targeting the pancreatic islet cells survive [16]. Destruction of islet beta cells by ICIs leads to insulin deficiency and consequently leads to the development of T1DM [17]. Most cases are caused by anti-PD-1 mono-therapy [18]. In contrast, some reports suggest that T1DM is more likely to occur with combination therapy (anti-CTLA-4 and anti-PD-1/anti-PD-L1)[17]. Although the frequency of T1DM due to anti-PD-L1 monotherapy is low, the present case suggests that careful observation should be needed [18]. Moreover, the data on the appropriate observation period after the last dose of ICIs are lacking. Immune checkpoint inhibitors affect memory T cells in the blood for more than 20 weeks after the final dose [19]. Considering the evidence, past reports, and the present case, we propose that a follow-up of at least six months is required.

## Conclusions

Severe irAE can occur six months after the discontinuation of ICI administration in ED-SCLC. Some patients with ED-SCLC, such as those with NSCLC, develop irAEs and have persistent durable responses. The appropriate observation period after the last dose of ICI is unknown. We should observe to detect and overcome irAEs, irrespective of histological type.

## References

[1] Goldman JW, Dvorkin M, Chen Y (2021). Durvalumab, with or without tremelimumab, plus platinum-etoposide versus platinum-etoposide alone in first-line treatment of extensive-stage small-cell lung cancer (Caspian): updated results from a randomised, controlled, open-label, phase 3 trial. Lancet Oncol.

[2] Liu SV, Reck M, Mansfield AS (2021). Updated overall survival and PD-L1 subgroup analysis of patients with extensive-stage small-cell lung cancer treated with atezolizumab, carboplatin, and etoposide (IMpower133). J Clin Oncol.

[3] Socinski MA, Jotte RM, Cappuzzo F (2023). Association of immune-related adverse events with efficacy of atezolizumab in patients with non-small cell lung cancer: pooled analyses of the phase 3 IMpower130, IMpower132, and IMpower150 randomized clinical trials. JAMA Oncol.

[4] Huang X, Yang M, Wang L, Li L, Zhong X (2021). Sintilimab induced diabetic ketoacidosis in a patient with small cell lung cancer: a case report and literature review. Medicine (Baltimore).

[5] Ricciuti B, Naqash AR, Naidoo J (2020). Association between immune-related adverse events and clinical outcomes to programmed cell death protein 1/programmed death-ligand 1 blockade in SCLC. JTO Clin Res Rep.

[6] Yokoo K, Kitamura Y, Suzuki K (2023). Relationship between immune-related adverse events and treatment effectiveness in extensive-disease small-cell lung cancer. Thorac Cancer.

[7] Song SO, Yun JS, Ko SH (2022). Prevalence and clinical characteristics of fulminant type 1 diabetes mellitus in Korean adults: a multi-institutional joint research. J Diabetes Investig.

[8] Moreau C, Drui D, Arnault-Ouary G, Charbonnel B, Chaillous L, Cariou B (2008). Fulminant type 1 diabetes in Caucasians: a report of three cases. Diabetes Metab.

[9] Le Lepvrier AL, Leysour de Rohello F, Brunel V, Prevost G (2020). Fulminant type 1 diabetes: report of a new French Caucasian case and recent findings. Diabetes Metab.

[10] Hatayama S, Kodama S, Kawana Y (2022). Two cases with fulminant type 1 diabetes that developed long after cessation of immune checkpoint inhibitor treatment. J Diabetes Investig.

[11] Imagawa A, Hanafusa T, Awata T (2012). Report of the Committee of the Japan Diabetes Society on the research of fulminant and acute-onset type 1 diabetes mellitus: new diagnostic criteria of fulminant type 1 diabetes mellitus (2012). J Diabetes Investig.

[12] Nishioki T, Kato M, Kataoka S, Miura K, Nagaoka T, Takahashi K (2020). Atezolizumab-induced fulminant type 1 diabetes mellitus occurring four months after treatment cessation. Respirol Case Rep.

[13] Garassino MC, Gadgeel S, Speranza G (2023). Pembrolizumab plus pemetrexed and platinum in nonsquamous non-small-cell lung cancer: 5-year outcomes from the phase 3 KEYNOTE-189 study. J Clin Oncol.

[14] Novello S, Kowalski DM, Luft A (2023). Pembrolizumab plus chemotherapy in squamous non-small-cell lung cancer: 5-year update of the phase III KEYNOTE-407 Study. J Clin Oncol.

[15] Stamatouli AM, Quandt Z, Perdigoto AL (2018). Collateral damage: insulin-dependent diabetes induced with checkpoint inhibitors. Diabetes.

[16] Clotman K, Janssens K, Specenier P, Weets I, De Block CE (2018). Programmed cell death-1 inhibitor-induced type 1 diabetes mellitus. J Clin Endocrinol Metab.

[17] Chen X, Affinati AH, Lee Y (2022). Immune checkpoint inhibitors and risk of type 1 diabetes. Diabetes Care.

[18] de Filette JM, Pen JJ, Decoster L (2019). Immune checkpoint inhibitors and type 1 diabetes mellitus: a case report and systematic review. Eur J Endocrinol.

[19] Osa A, Uenami T, Koyama S (2018). Clinical implications of monitoring nivolumab immunokinetics in non-small cell lung cancer patients. JCI Insight.

